# Click-Chemistry-Enabled Functionalization of Cellulose Nanocrystals with Single-Stranded DNA for Directed Assembly

**DOI:** 10.1021/acsbiomaterials.4c01518

**Published:** 2024-09-11

**Authors:** Daria Bukharina, Katherine Cauffiel, Laura Mae Killingsworth, Justin A. Brackenridge, Valeriia Poliukhova, Minkyu Kim, Justin Brower, Julio Bernal-Chanchavac, Nicholas Stephanopoulos, Vladimir V. Tsukruk

**Affiliations:** † School of Materials Science and Engineering, Georgia Institute of Technology, Atlanta, Georgia 30332, United States; ‡ Department of Chemical Engineering, Dankook University, Yongin 16890, Republic of Korea; § School of Molecular Sciences, Arizona State University, Tempe, Arizona 85281, United States; ∥ Biodesign Center for Molecular Design and Biomimetics, Arizona State University, Tempe, Arizona 85251, United States

**Keywords:** cellulose nanocrystal chiral complexation, DNA-mediated self-assembly, cellulose nanocrystal chiral grafting, CNC surface functionalization

## Abstract

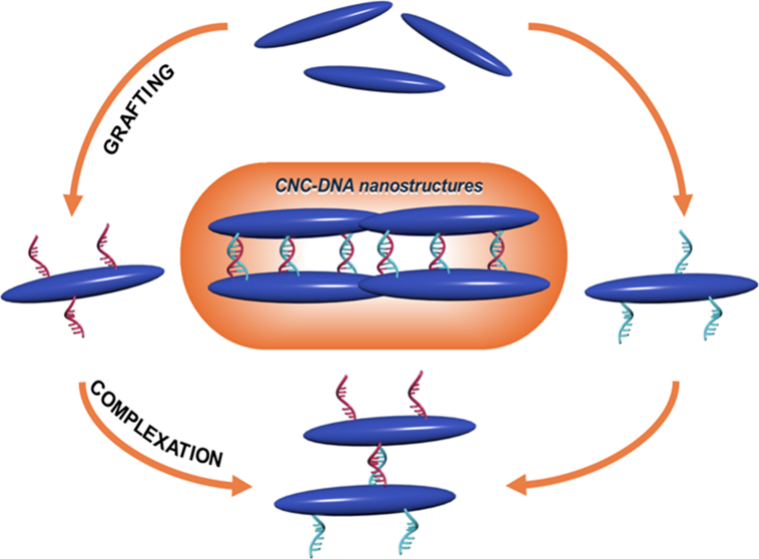

Controlling the self-assembly of cellulose nanocrystals (CNCs) requires precise control over their surface chemistry for the directed assembly of advanced nanocomposites with tailored mechanical, thermal, and optical properties. In this work, in contrast to traditional chemistries, we conducted highly selective click-chemistry functionalization of cellulose nanocrystals with complementary DNA strands via a three-step hybridization-guided process. By grafting terminally functionalized oligonucleotides through copper-free click chemistry, we successfully facilitated the assembly of brushlike DNA-modified CNCs into bundled nanostructures with distinct chiral optical dichroism in thin films. The complexation behavior of grafted DNA chains during the evaporation-driven formation of ultrathin films demonstrates the potential for mediating chiral interactions between the DNA-branched nanocrystals and their assembly into chiral bundles. Furthermore, we discuss the future directions and challenges that include new avenues for the development of functional, responsive, and bioderived nanostructures capable of dynamic reconfiguration via selective complexation, further surface modification strategies, mitigating diverse CNC aggregation, and exploring environmental conditions for the CNC–DNA assembly.

## 1 Introduction

Cellulose nanocrystals (CNCs) are 1D nanostructures commonly derived from plant celluloses through mineral acid hydrolysis.^1^ These nanocrystals exhibit unique mechanical and photonic properties, such as high strength and stiffness combined with selective polarized light reflection.^2−4^ The properties are the direct result of CNC self-assembly into long-range hierarchical helical structures upon reaching a critical concentration and entering a lyotropic liquid crystal phase.^2,4,5^

Characteristic Bouligand structures with twisted helical organization at mesoscale are formed in thin films upon slow solvent evaporation,^6^ a phenomenon called evaporation-induced self-assembly (EISA). In such an organization, each successive layer of ordered nanocrystals is rotated with respect to the previous one at a specific angle, forming a chiral nematic arrangement with submicron helical pitch length.^7^ This self-assembly of CNCs can be harnessed to create functional nanocomposites incorporating other materials, thereby expanding the functionalities obtained. Enhancing the control over the arrangement and alignment of individual nanocrystals can tune mechanical, thermal, and chiroptical properties;^5^ but achieving these pathways requires inducing and fundamental understanding of the selective CNC interactions with surrounding, as well as the chemistry at their interfaces, and any resultant hierarchical structures that arise from these interactions.^8^ The precise control of CNC characteristics is crucial across a range of applications, from biomedical implants to coatings, sensors, and electronic devices.^2,3,9,10^

The functionalization of CNCs is a critical step for suspension stabilization and facilitating their successful self-assembly. The surface of nanocelluloses is mostly composed of hydroxyl groups, arising from anhydroglucose rings linked through β-1-4 glucoside-l,^5^ that can be chemically modified via synthetic processes. Initial surface modification happens after acid-catalyzed hydrolysis, during which amorphous regions of the cellulose degrade, leaving crystalline parts intact; for example, the use of sulfuric acid leads to nanocrystals with a negative surface charge due to the incorporation of sulfate groups. CNCs with carboxyl, hydroxyl, catechol, sulfonate, phosphate, or pyridyl functionalities can be prepared via TEMPO oxidation,^11^ alkali desulfation,^12^ chlorosulfuric acid treatment,^13^ and phosphorylation with sodium dihydrogen phosphate dihydrate and sodium hydroxide^14^ and 4-chloropyridine with potassium hydroxide,^15^ yielding functionalized nanostructures that can potentially further be modified with oligonucleotides or peptides.^16^ However, the direct surface modification of cellulose nanocrystals presents significant challenges, primarily due to the potential loss of colloidal stability and the disruption of their intrinsic helical organization.

Therefore, there have been only a handful of reports of grafting biomaterials onto CNCs due to the complexity of the grafting procedures and the need for their prefunctionalization. Among biomaterials is deoxyribonucleic acid (DNA) with nucleotides encoded by specific base-pairing interactions (A with T, and G with C)^17^ to form a double helix.^18^ These selective interactions have been utilized in DNA-guided assembly to fabricate versatile bioderived nanostructures for precisely guided interactions. The programmability of DNA hybridization allows for directed assembly strategies to create structures of controlled size (1–100 nm), with preprogrammed organization.^19^ For example, DNA origami^20^ utilizes hundreds of short synthetic DNA strands to create various monodisperse nanoscale shapes. 3D DNA origami was further demonstrated by folding DNA multilayered helices into a honeycomb lattice,^21^ complex nanoparticles,^22^ and complementary pairing.^23^ A variety of different crystal symmetries can be designed by altering the strength of DNA hybridization, achieving stability and responses to stimuli.^24,25^ Overall, DNA-guided assembly is driven by predictable and controllable interactions and specific chemistry with high precision at the nanoscale advancing bottom-up nanostructures’ fabrication^22^ and reversible interactions.^23^

To date, DNA has been coupled to cellulose polysaccharides for various purposes, such as mRNA purification^26^ and asymmetric catalysis.^27^ DNA-modified cellulose nanostructures were first synthesized by Naylor and Gilham,^28^ and DNA was attached to a solid cellulose matrix.^29^ Astell and co-workers linked DNA to a cellulose paper by phosphate ester formation.^30^ In another work, UV irradiation allowed for double-stranded DNA immobilization onto a nonwoven cellulose fabric for trapping heavy metal ions^31^ or pollutants.^32^

However, selective grafting of complementary DNA strands onto cellulose nanocrystals and controlling their assembly have been rarely reported to date. To the best of our knowledge, the only DNA-grafted CNCs have been reported by Mangalam et al., where they implemented EDC coupling to conjugate DNA to the CNCs surface and demonstrated some difference in assembling behavior, however, the effect on the chiroptical properties has not been studied.^33^ Although effective, EDC coupling is generally less efficient than click reactions, especially in the presence of water where the intermediate components can hydrolyze.^34^ In contrast, click reaction, which can be conducted under mild conditions, is more versatile for bioconjugation and other applications where precise coupling is required with high efficiency, specificity, yields, and biorthogonality.^35^

It is critically important that click chemistry allows CNC surface modification under mild conditions (room temperature, various solvents, including organic and/or aqueous solvents, large pH range of 4–11). Previously, introduction of click-functionalized nanoparticles into polymer matrices has led to the development of self-healing materials with improved durability and performance^36^ or was used to cross-link polymers and nanoparticles within hydrogels, resulting in nanocomposites with enhanced mechanical properties.^37^ It is worth noting that click chemistry, although vastly applied to functionalize various nanostructures from nanoparticles and quantum dots to polymers,^38−40^ has not yet been explored for CNC functionalization.

Thus, in this work, we report a click-chemistry synthetic route for obtaining functionalized cellulose nanocrystals and their further grafting with complementary single-stranded DNA (ssDNA) handles. We exploited a highly selective three-step process that allowed for hybridization-guided self-assembly of the nanocrystals with distinct chiroptical properties. This study demonstrates the feasibility of utilizing click chemistry^41^ for CNC bio-functionalization with different grafting habits. Furthermore, we demonstrate that ssDNAs are able to form chiral complexes on the surface of CNCs and have the potential to affect the final assembly of the nanocrystals in functional films.

**Figure 1:**
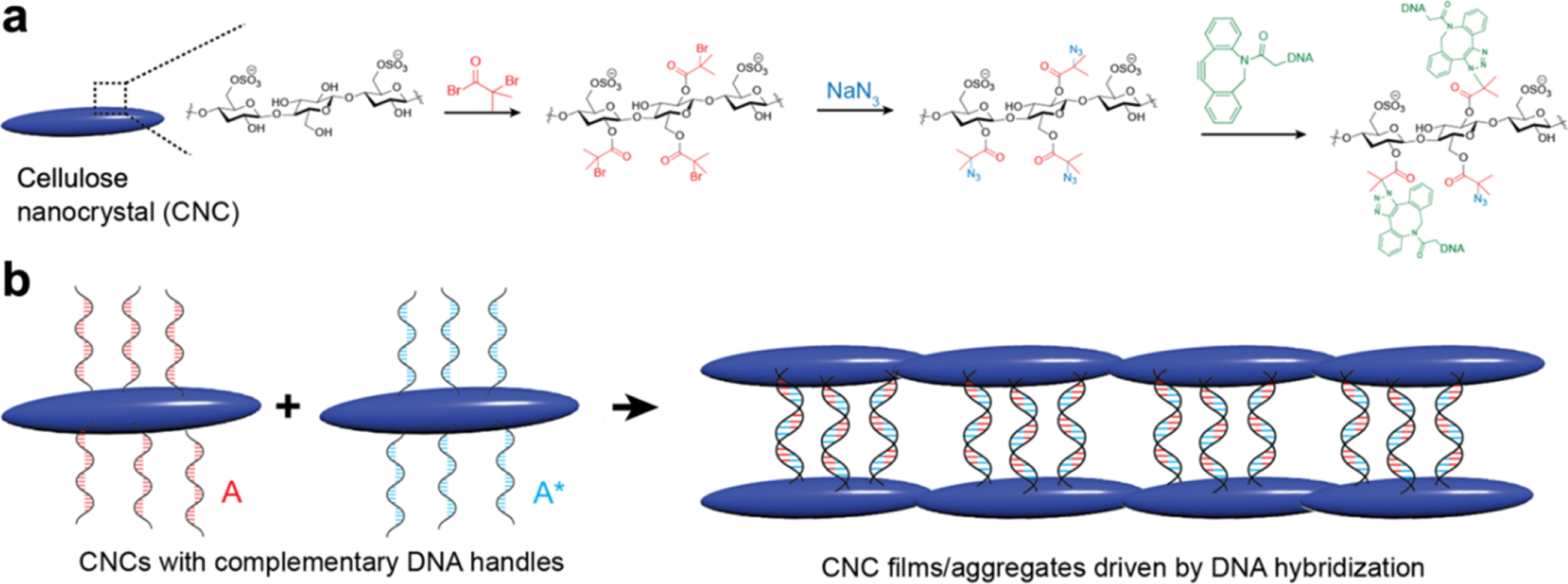
Schematic of (a) the surface modification steps implemented in this work and (b) DNA complexation of the complementary DNA strands grafted on the CNC surface and the final CNC assembly morphology.

In this approach, first, terminal surface hydroxyl (–OH) groups were esterified by bromoisobutyryl bromide providing bromine (–Br) surface groups,^42,43^ which were then converted to azide (–N_3_) groups to facilitate click chemistry^44,45^ (Figure 1).

In the final step, to graft oligonucleotides onto the CNC surface, the azide surface groups were reacted with dibenzocyclooctyne (DBCO)-modified oligonucleotides via strain-promoted azide-alkyne cycloaddition (colloquially known as “copper-free click”).^42,43^

## 2 Materials and Methods

### 2.1 Materials

2-Bromoisobutyryl bromide (BIBB) (purity > 98.0%(GC)) and sodium azide (NaN_3_) (>99.0%) were purchased from TCI America. Triethylamine (TEA), used for the synthesis, was purchased from Millipore Sigma. 4-(Dimethylamino)pyridine (DMAP) ReagentPlus (≥99% purity) and high-quality ACS grade dimethylformamide (DMF) (99.96%) were purchased from Sigma Aldrich.

### 2.2 CNC Synthesis

CNCs studied here were isolated from wood pulp by acid hydrolysis according to an established protocol.^46^ Briefly, 5 g of dried wood pulp pieces were added to 95 g of 64 wt % sulfuric acid solution at 45 °C and stirred continuously for an hour. To quench hydrolysis, the reaction solution was poured into a glass container of 10-fold acid volume ultrapure water. The suspension was incubated overnight to enable phase separation, and the bottom layer was decantated and centrifuged for 10 min at 4300 rcf to remove unhydrolyzed products from resultant CNCs.

After the washing steps, the supernatant was exchanged with ultrapure water. Then, the CNC suspension was dialyzed against water until the pH was neutral. In order to obtain a homogeneous and well-dispersed suspension, the resulting suspension was centrifuged again at 14 500 rcf and then tip-sonicated at 40% amplitude 5 s on/5 s off for 4 min and 30 s using a large tip sonicator (Qsonica Q125 with 1/8 inch diameter probe).

### 2.3 Bromination of CNCs (CNC-Br)

To complete the first step of our synthesis process and substitute the CNC hydroxyl groups with bromine, we conducted solvent exchange for CNCs from an aqueous solution to DMF. CNCs in water were placed in dialysis tubing and dialyzed against DMF over several days, changing the dialysis media every 12 h for a total of eight DMF changes. This process is critical for preparing the CNCs for the subsequent bromination step, where DMF is a preferred solvent, facilitating interactions between components. In an aqueous solvent, CNCs will undergo a reaction with α-bromoisobutyryl bromide (BIBB) or hydrolysis of the ester bond formed between the CNC surface and BIBB in a basic environment, leading to the cleavage of the bromine-containing group and thereby undoing the chemical modification intended to enhance the CNCs’ functionality.^47^

After the solvent exchange was complete, 10 mL of CNCs in DMF (1 wt %) was added to 30 mL of DMF and placed in an ice bath. TEA (2.6 mL) was added to CNCs to adjust the pH, and 0.6 g of DMAP was added as a catalyst for the bromination reaction.^48^ The BIBB mixture in DMF (2 mL of BIBB + 5.5 mL of DMF) was slowly added dropwise to the CNC–TEA–DMAP mixture and stirred vigorously for 10 min. After this, the ice bath was removed and the mixture was allowed to react for 24 h. The reaction product was purified by dialysis against DMF.

### 2.4 Substitution of Bromine with Azide Groups (CNC-N_3_)

The purified CNC-Br product (0.8 wt %) underwent a reaction with sodium azide (NaN_3_) to substitute the bromine on the CNC surface with azide groups, and an obtained product was called CNC-N_3_. Forty milliliters of CNC-Br in DMF was transferred to a flask and placed in an oil bath at 50 °C, and 170 mg of NaN_3_ was slowly added to the mixture. The mixture was allowed to react for 48 h with vigorous stirring. (Caution: sodium azide decomposes at 275 °C, and rapid heating above this temperature can cause decomposition and explosion. To avoid gas accumulation, the flask stopper was punctured with a needle.) The reaction product was purified by dialysis against DMF or water (caution: sodium azide reacts with water to form hydrazoic acid, a highly toxic and explosive gas).

### 2.5 Synthesis of CNC-Oligonucleotides

For single-stranded DNAs or oligonucleotides synthesis, unpurified amine-modified oligonucleotides were purchased from IDT at a 1 μM scale. The strands were modified with a ∼2.63× excess of the sulfo-NHS-DBCO reagent (dissolved in anhydrous DMSO in 20 mM aliquots) relative to DNA in 1× PBS at pH 7.4. Each strand was agitated overnight at room temperature before being washed with nanopure water in a 3 kDa molecular weight cutoff filter (MWCO). The strand was then purified on an Agilent reverse-phase HPLC (RP-HPLC) with a linear gradient of 0–100% of 50 mM TEAA/methanol via an Agilent AdvanceBio Oligonucleotide column. After purification, the samples were washed with nanopure water and concentrated using 3 kDa MWCO filters.

The synthesized oligonucleotides 1 and 2 (named O_1_ and O_2_) are:
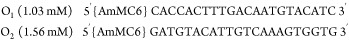
where {.AmMC6} indicates 5′ C6 amino linkers.^49,50^

To modify CNC-N_3_ with oligonucleotides,^51^ 50 μL of the DBCO-modified oligonucleotides were added to 5 mL of CNC-N_3_ (0.68 wt %) and allowed to react for 24 h before purification via dialysis. The CNCs modified with complementary strands (synthesized separately) were then added together in a 1:1 volume ratio and allowed to self-assemble by mixing them for 24 h as described further.

### 2.6 DNA-Led Complexation of CNCs

To induce DNA complexation between nanocrystals, the two suspensions of CNCs grafted with complementary oligonucleotides were mixed in a 1:1 volume ratio in the presence of NaCl (the resultant concentration of which was 50 mM). The resulting mixture was kept at 40 °C for 15 h and then cooled before the experiments.

### 2.7 Characterization Methods

#### 2.7.1 Attenuated Total Reflectance Fourier Transform Infrared Spectroscopy

Attenuated total reflectance Fourier transform infrared spectroscopy (ATR-FTIR) measurements were conducted in transmission mode to monitor the chemical composition of chemically grafted CNCs and their molecular interactions in assembled films using a Bruker Vertex 70 system with a resolution of 1 cm^–1^ and a number of scans of 100. For each sample, 200 background scans on a silicon ATR crystal without the sample were collected before sample deposition.

#### 2.7.2 UV–vis Spectroscopy

A Shimadzu UV-3600 Plus spectrometer was used to collect the absorbance spectra of the CNC suspensions within the 180–600 nm range. Measurements were conducted in a quartz cuvette with a 10 mm light path. Background measurements of DMF were recorded prior to data collection.

#### 2.7.3 X-ray Photoelectron Spectroscopy

X-ray photoelectron spectroscopy (XPS) was used to measure the surface elemental composition and chemical and electrical state of the materials. The spectra were acquired using a Thermo Scientific Nexsa G2 X-Ray photoelectron spectrometer equipped with an Al Kα monochromate microfocused source, with a spot size of 400 μm. The survey scan spectra were collected three times with binding energies of 0–1350 eV in steps of 1 eV. The high-resolution scans were collected 10 times in 0.1 eV steps. Thermo Scientific Avantage Software was used for acquiring the data and processing it.

#### 2.7.4 ζ-Potential Measurements

ζ-Potential was measured with Zetasizer Nano ZSP (Malvern Instruments) in polystyrene cuvettes and with the Smoluchowski model. The ζ-potential is derived from the electrophoretic mobility of the CNCs, which was determined from electrophoretic light scattering (ELS). For each sample, the ζ-potential value was reported as an average of three runs, where each run was the average of 20 measurements.

Measurements in DMF were attempted for CNC-Br and CNC-N_3_; however, DMF is incompatible with the electrode, resulting in their damaging and compromised values. Thus, for CNC-N_3_, after successful modification of the bromine groups, the solvent exchange to water was conducted by completing the purification steps through dialysis against water. O_1_ and O_2_ were added to CNC-N_3_ in water in order to measure the surface charge of the obtained CNC-O_1_ and CNC-O_2_.

#### 2.7.5 Atomic Force Microscopy

Atomic force microscopy (AFM) imaging was carried out to observe the surface morphology of samples using an ICON Dimension microscope (Bruker) in the light tapping mode.^52^ Samples were drop cast as well as spin cast (at 3000 rpm for 30 s) onto freshly piranha-treated silicon wafers. AFM probes purchased from Mikro-masch (Hi’Res-C15/Cr-Au and HQ:XSC11/AL BS) were used with a desired spring constant depending on the stiffness of the samples.

The scanning rate varied in the range of 0.6–1.0 Hz, based on the scan size. The resolution of the AFM images was either 512 × 512 pixels or 1024 × 1024 pixels. All AFM images were processed and analyzed using Nanoscope Analysis software (Bruker) or Gwyddion Software. The tip radii for the probes used in this work were 8 and 2 nm, respectively, for Hi’Res-C15/Cr-Au and HQ:XSC11/AL BS. For high-resolution AFM, Micro Mash HiRes-C18/Cr-Au tips (2.8 N/m) with a radius of ∼2 nm were used.

The linear roughness (*R*_a_) was calculated for the surface of pristine and oligonucleotide-modified CNCs along the reference line of identical length of the main nanocrystal axis in NanoScope software with the Section command.

#### 2.7.6 Ellipsometry

An M-2000U spectroscopic ellipsometer with WVASE32 was used to measure Mueller Matrices (MM) to obtain circular birefringence (CB) spectra in the transmission mode with components normalized with respect to m_11_.^53^ As known, MM spectroscopy measures the 16 elements of the polarization transfer matrix. In this analysis, the polarization state of light can be described by a four-element Stokes vector *S*. A 4 × 4 matrix describes how a sample modifies the polarization state of the incoming light (described by *S*_in_) to the outcoming Stokes vector *S*_out_ = *MS*_in_.

For nondepolarizing samples, the Mueller matrix can be related to circular dichroism and birefringence, **CD** and **CB**, as well as the horizontal and 45° projections of linear dichroism (**LD** and **LD′**) and linear birefringence (**LB** and **LB′**) as:^54^
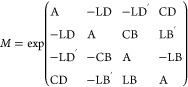
1where different elements are noted as: m_11_,_22_,_33_,_44_ = A, m_12_,_21_ = −LD, m_13_,_31_ = −LD’, m_14_,_41_ = CD, m_23_ = CB, m_24_ = LB’, m_32_ = −CB, m_34_ = −LB, m_42_ = −LB’, m_43_ = LB.

CB indicates the difference in the speeds of the propagation of left- and right-handed circular polarization states, whereas the CD measures a sample’s selectivity for the transmission of left- and right-handed circular polarization.

#### 2.7.7 Circular Dichroism Measurements

Circular dichroism (CD) was measured with a JASCO J-815 spectropolarimeter by drop-casting assembled nanocrystals onto a quartz slide. A background measurement of a clean quartz slide was recorded prior to sample measurements and subtracted from the measurements.

#### 2.7.8 Polarized Optical Microscopy (POM)

An Olympus BX51 optical microscope was used to characterize the visual appearance of CNC films in the bright mode. Additionally, the dark-field (DF) mode was used to visualize the optical activity of the modified nanocrystals due to their anisotropy and birefringence. The Fiber-Lite DC-950 light source was used.

## 3 Results and Discussion

### 3.1 Initial Surface Functionalization of CNCs

**Figure 2:**
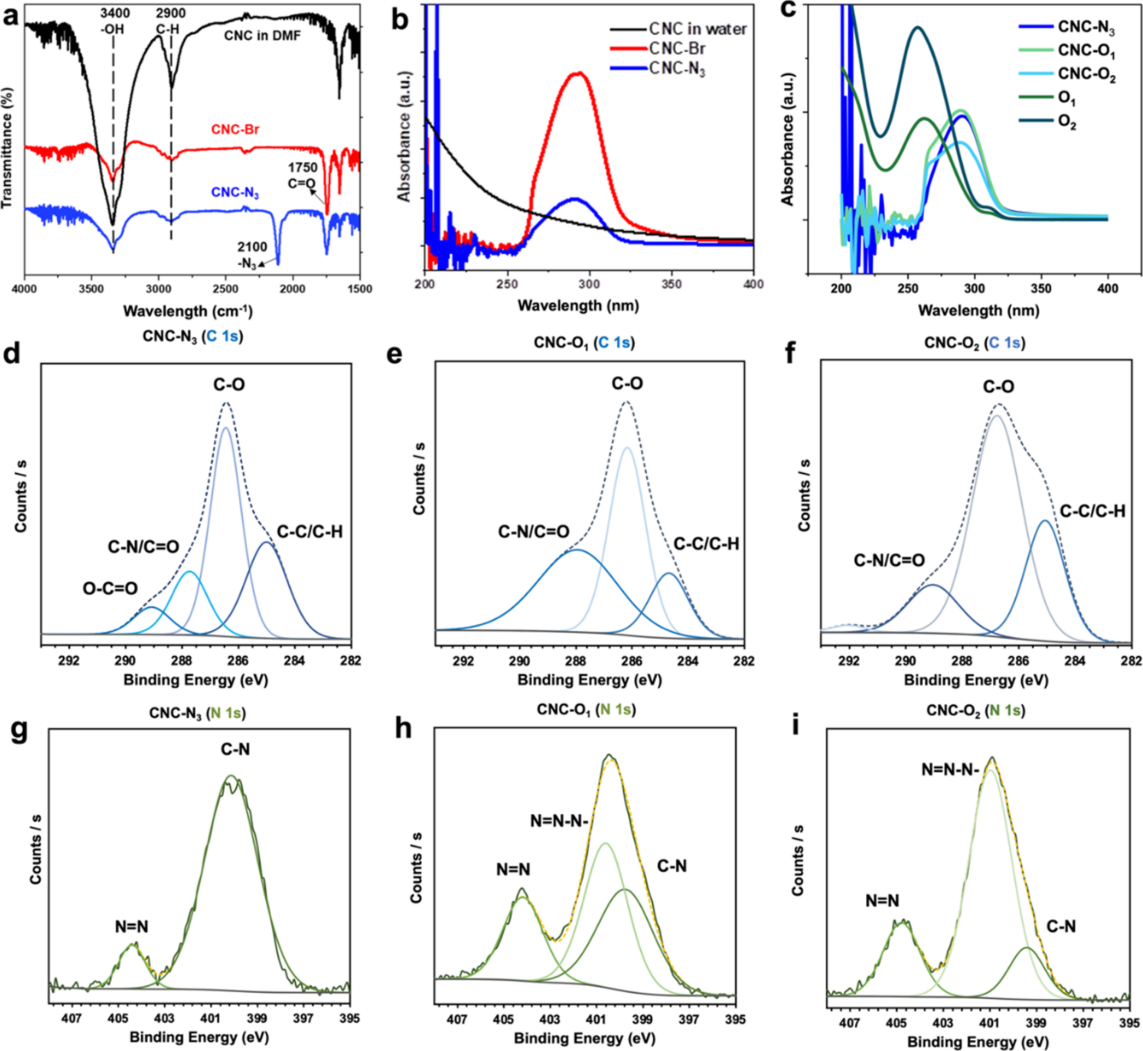
(a) FTIR transmittance spectra of CNCs in DMF and modified CNC-Br and CNC-N_3_. UV–vis absorbance spectra of (b) unmodified CNCs in water and modified CNC-Br and CNC-N_3_. Note that unmodified CNCs were measured in water because of the high absorbance of DMF below 250 nm. (c) UV-vis absorbance spectra of azide-modified CNCs (CNC-N_3_) and CNCs modified with complementary oligonucleotides (CNC-O_1_ and CNC-O_2_), as well as spectra for O_1_ and O_2_ in water. XPS narrow high-resolution scan of the C 1s and N 1s regions for (d, g) CNC-N_3_, (e, h) CNC-O_1_, and (f, i) CNC-O_2_.

The reaction with α-bromoisobutyryl bromide (BIBB) in DMF was catalyzed by 4-dimethylaminopyridine (DMAP) and pH-stabilized by triethanolamine (TEA) (Figure 1a).^55,56^ Successful bromination was confirmed by the appearance of a new FTIR peak at 1750 cm^–1^ corresponding to the C=O from BIBB (Figure 2a).^56,57^

Next, to substitute bromine with an azide group, CNC-Br was reacted with sodium azide. Following the dialysis of the reaction mixture and the removal of excess unreacted reagents, the grafting of the –N_3_ groups on the surface of CNCs was confirmed by observing an appearance of a high-intensity peak on the FTIR spectrum at 2100 cm^–1^ (corresponding to N_3_ bond stretching) (Figure 2a).^58^ Finally, an absorbance peak at 290 nm on the UV–vis spectrum was observed for both bromine- and azide-terminated CNCs (Figure 2b) due to the carbonyl (C=O) group^59^ from BIBB and an azide group,^60^ both of which absorb UV light at 290 nm. In conclusion, all of these results together confirm successful surface functionalization with both α-bromoisobutyryl bromide and azide.

**Figure 3:**
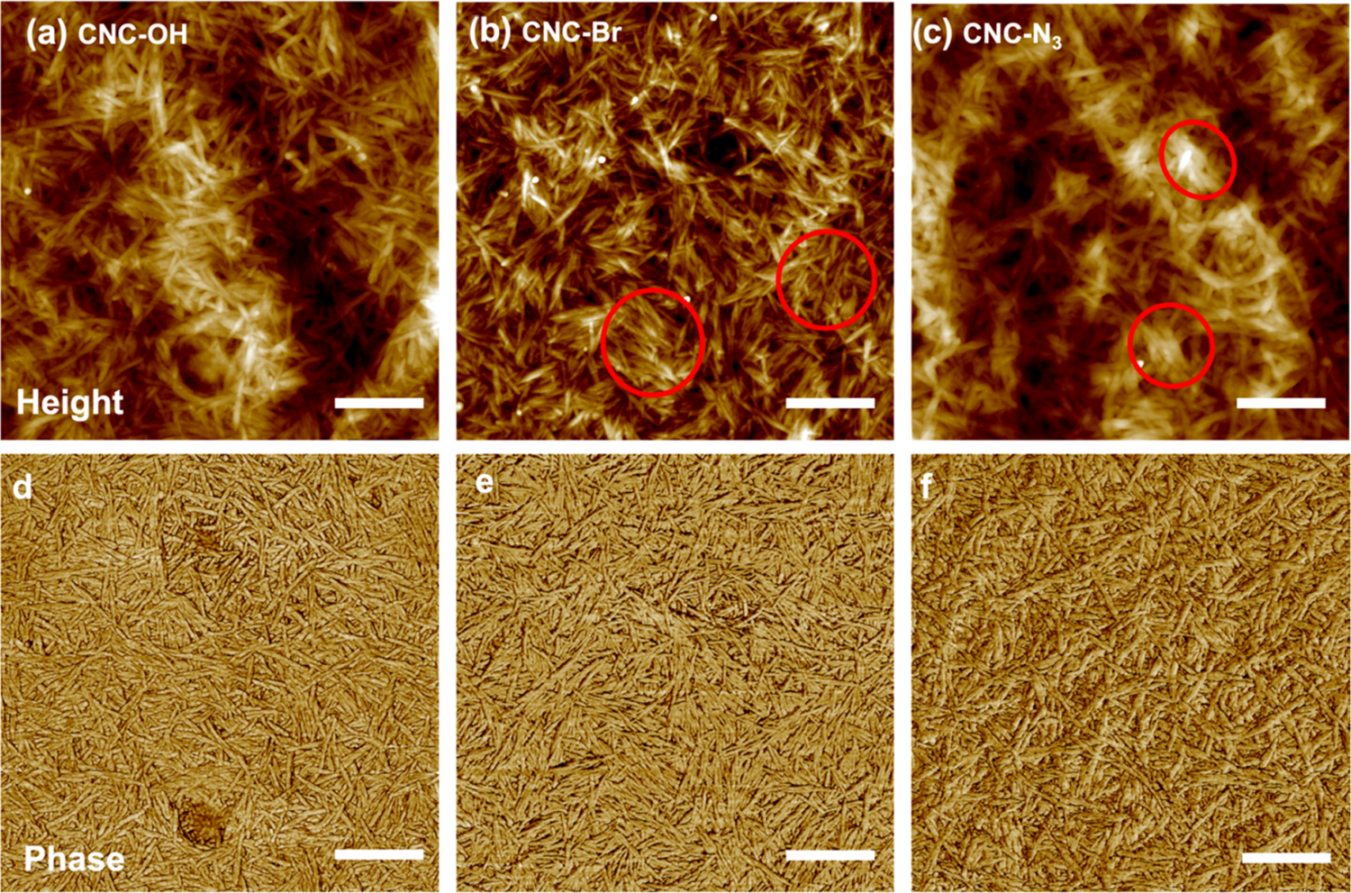
AFM topography images of (a) CNC-OH in DMF, (b) CNC-Br, (c) CNC-N_3_, and the corresponding phase images (d–f). All scale bars are 400 nm. Samples were drop cast onto a Si wafer. Red circles visualize local bundles of nanocrystals.

Furthermore, to examine the morphology of modified needle-like CNCs, high-resolution AFM imaging was performed after each modification step and compared with pristine nanocrystals (Figure 3).

In all cases, the nanocrystals did not appear highly oriented on a sub-micrometer scale and were rather randomized due to the partial aggregation of CNCs in organic solvents such as DMF during film assembly.^61^ However, even partially aggregated CNCs in organic solvents were available for organization on a nanoscale. Local uniform orientation of bundles of tens of rightly packed nanocrystals can be observed after different modification steps (Figure 3a–c).

Additionally, the as-prepared suspensions appeared translucent and exhibited pale blue coloration, a shade typical for stable colloidal CNC suspensions (Figure S1). Colloidal stability can be evaluated not only by the presence of particle aggregation but also by the surface charge of the nanocrystals in the suspensions.

Thus, the ζ-potential of the as-modified CNC suspensions was measured for the evaluation of the suspension stability and changes in surface chemistry after the reactions. CNCs prepared by sulfuric acid hydrolysis had a negative surface charge and a ζ-potential value of −52 mV (Table S1).^62^ While bromine groups are also negatively charged and the ζ-potential of CNC-Br recorded in the negative values, the actual absolute values were affected by the DMF solvent reactivity with the surface of electrodes and registered lower values (−12.8 eV). In the case of CNC-N_3_, the ζ-potential measurements were possible to record with confidence after solvent exchange to water through the dialysis process. When compared to negatively charged unmodified CNCs in water (−50 mV), the negative surface charge of CNC-N_3_ was −19.9 mV. The reduction in the ζ-potential absolute values was due to the substitution of the hydroxyl groups on the CNCs surface and the grafting of neutrally charged –N_3_ groups.

### 3.2 Click-Chemistry-Mediated Grafting of Complementary Oligonucleotides

Next, copper-free click chemistry was utilized to perform an alkyne–azide cycloaddition between the azide groups on modified CNCs and dibenzocyclooctyne (DBCO)-terminated oligonucleotides (Figure 1a).^51^ Successful reaction completion was first confirmed by changes in the UV–vis absorbance spectra (Figure 2c). At this step, alkyne–azide cycloaddition between CNC-N_3_ suspended in either water or DMF was performed (Figure S2). The two complementary strands, DBCO-modified Oligo_1_ and Oligo_2_ (Figure S3), were added separately to CNC-N_3_ in DMF and measured after the dialysis of the reaction mixture was performed to remove any unconjugated DNA. One of the challenges at this step was to achieve complete surface modification; due to the excessive cost of modified DNA, only small volumes of the oligonucleotides could be obtained, thereby restricting the final product characterization.

Furthermore, partial modification could be due to the known aggregation of the modified CNCs in organic solvents, which made it a challenge to achieve complete substitution of the surface groups with oligonucleotides, as revealed in Figure 2c. Evidently, a modification of CNC-N_3_ with oligonucleotides 1 and 2 was achieved, as seen by the broader 290 nm peak and characteristic shoulder at 260 nm (Figure 2c). The 260 nm peak, indicating the presence of nucleic acids on the CNC surface due to the purine and pyrimidine bases,^63^ has not been observed for pristine CNCs, or the intermediate CNC-N_3_ (Figure 2b,c). Additionally, the ζ-potential in the aqueous suspensions increases in the absolute values for CNC-oligonucleotides (−27.2 mV) compared to the CNC-N_3_ (−19.9 mV, Table S1), which is consistent with the high negative charge of the DNA handles.^64^ FTIR spectra did not demonstrate significant differences because of peak overlapping (Figure S4). Thus, further surface chemistry analysis was pursued with XPS (Figure 2).

High-resolution XPS scans of the C 1s and N 1s regions confirm that the click reaction occured via triazole formation (as shown in Figure 1a) between the azide group on the CNCs surface and the alkyne of the DBCO-modified oligonucleotides. Indeed, triazole formation was indicated by the presence of the N 1s peak at ∼401 eV (corresponding to the N–N=N bond, Figures 1a and 2g–i).^65^ On the C 1s scans, the peak at 287.7 eV corresponds to the C–N/C=O peaks and is seen to transform into a broader 289 eV C=O peak after the reaction (Figure 2d–f). The survey XPS scans confirm that azide (–N_3_) groups were formed in 1 atom %, while 1.96 out of 2.16 atom % Br groups were substituted after the reaction (Figures S5 and S6). Then, we observed an increase in N% from 3.9 to 8.4%, in addition to the successful triazole bond formation, for CNC-O_1_ (Figure S6). By taking into account the number of nitrogen atoms in the O_1_ sequence (75 N atoms) and the increase in nitrogen content by 4.46%, we can conclude that approximately 5.6% of the CNCs surface was successfully grafted with ssDNA strands. Finally, from the CNC-O_2_ peak (72 N atoms) indicating a content increase of 4.76%, we can estimate that approximately 6.6% of the CNC surface was modified with O_2_.

### 3.3 Morphology of Individual and Modified Nanocrystals

**Figure 4:**
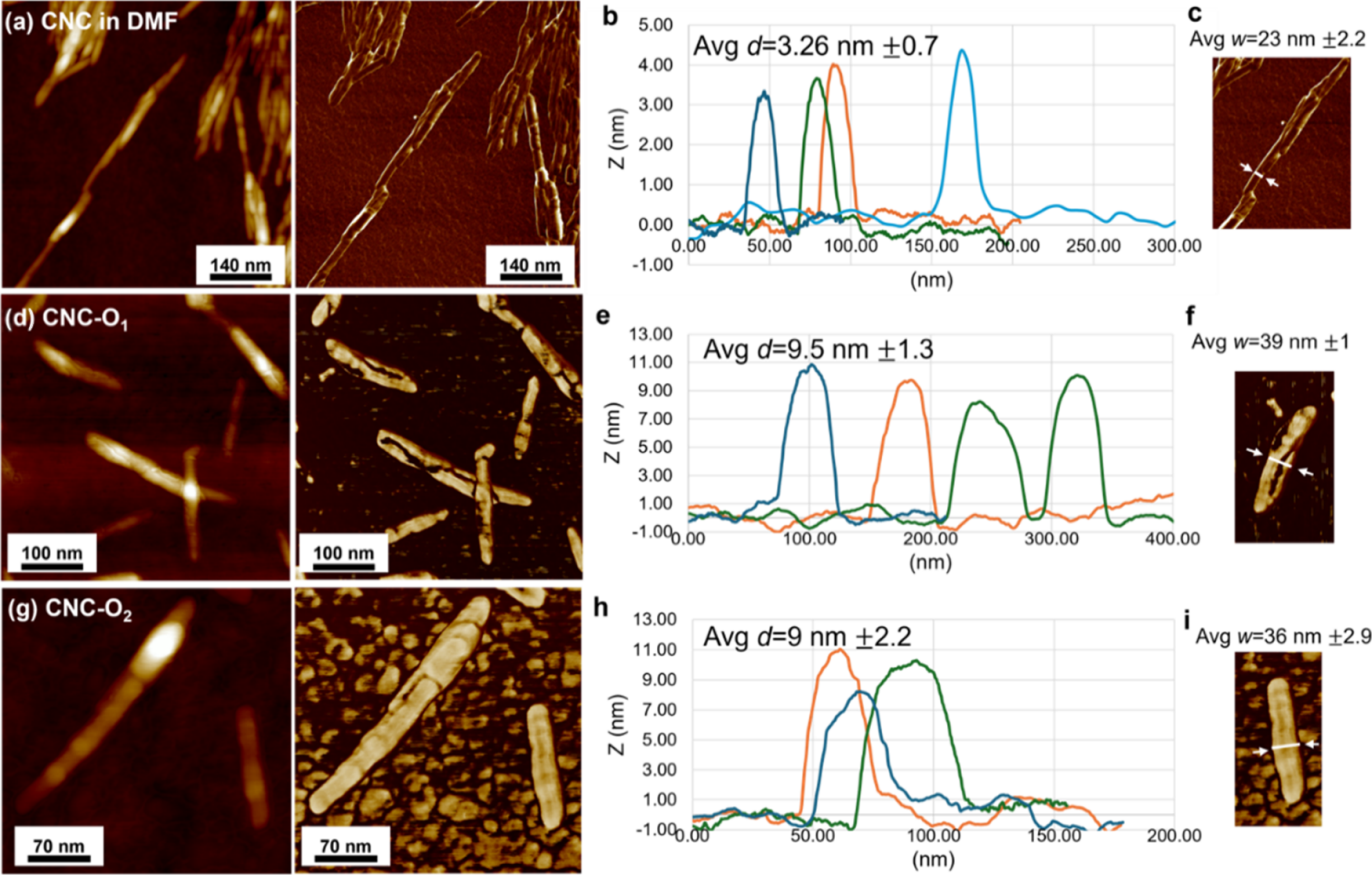
High-resolution AFM topography and phase scans of (a) CNC in DMF, (d) CNC-O_1_, and (g) CNC-O_2_. (b) CNC in DMF, (e) CNC-O_1_, and (h) CNC-O_2_ topography profiles obtained from the AFM topography images, with the average diameter (Avg *d*) of the CNCs corresponding to max *Z* height. (c, f, i) Apparent diameter from the phase images.

Next, we studied the assembly of ssDNA-modified CNCs as individual entities after suspension evaporation and adsorption and drying on an atomically flat substrate. First, for comparative analysis, unmodified CNCs were scanned and, as expected, individual high-aspect needles were observed on high-resolution images (Figure 4a).

The average pristine nanocrystal diameter was obtained from the multiple topographical profiles as 3.3 ± 0.7 nm (Figure 4b,c). Their apparent diameter was around 20 nm and includes some tip dilation as well-known for imaging nanostructures.^52^ Notably, their phase image demonstrated low contrast relative to the substrate due to similar surface stiffness and adhesion of cellulose nanocrystals and silicon dioxide surface.^66,67^

In contrast, the phase images demonstrated high contrast between modified nanocrystals and the substrate, immediately confirming changes in their surface chemistry (Figure 4a–g). For example, Figure S7c visualizes the partial grafting of the CNCs with oligonucleotides, compared to predominantly observed uniformly covered nanocrystals (Figures 4g and S8). It is known that depending on the properties of the surface, such as adhesive attraction or mechanical compression (stiffness), the interaction between the tip and surface will cause a phase shift and, therefore, result in high phase image contrast.^68,69^ Thus, from the phase images, one can see how the surface of modified nanocrystals is wrapped with materials of different stiffness, causing phase lag and high contrast with very hard Si substrate. This is an indication that the oligonucleotides are indeed successfully grafted on the CNC surface as their Young’s modulus is around 0.3–1 GPa,^70^ 2 orders of magnitude lower than that of the silicon substrate. Next, in oligonucleotide-modified CNCs, individual nanocrystals appeared significantly larger in diameter. However, due to the complicated tip dilation effect, the quantitative analysis here is based on reliable height measurements from the topographical profiles.

In fact, from the profile analysis, the average diameter of a modified CNC dramatically increased (almost tripled) to 9.25 ± 2.7 nm compared to pristine nanocrystals (Figure 4e,f). This dramatic increase in nanocrystal diameter indicates the presence of additional grafted materials. The grafting density can be evaluated from the coating thickness (3 nm) and molecular dimensions of DNA strands. The length of the 22-base pair long oligonucleotides can be estimated to be 15 nm in a fully extended state, considering the 0.68 nm length of one base pair and the single-strand diameter of ∼1 nm.^71,72^

From these data, the grafted chains density, ∑ (chains/nm^2^), can be estimated according to eq 2(73)
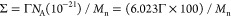
2where *N*_A_ is Avogadro’s number, *M*_n_ (g/mol) is the number average molar mass of grafted oligonucleotides (taken as 6817.5 and 7008.6 g/mol, for O_1_ and O_2_, respectively), and Γ (mg/m^2^) is the surface coverage taken as [Γ = thickness of the layer (3 nm) × density of attached DNA strand (1.1 g/cm^3^)].^73^

**Figure 5:**
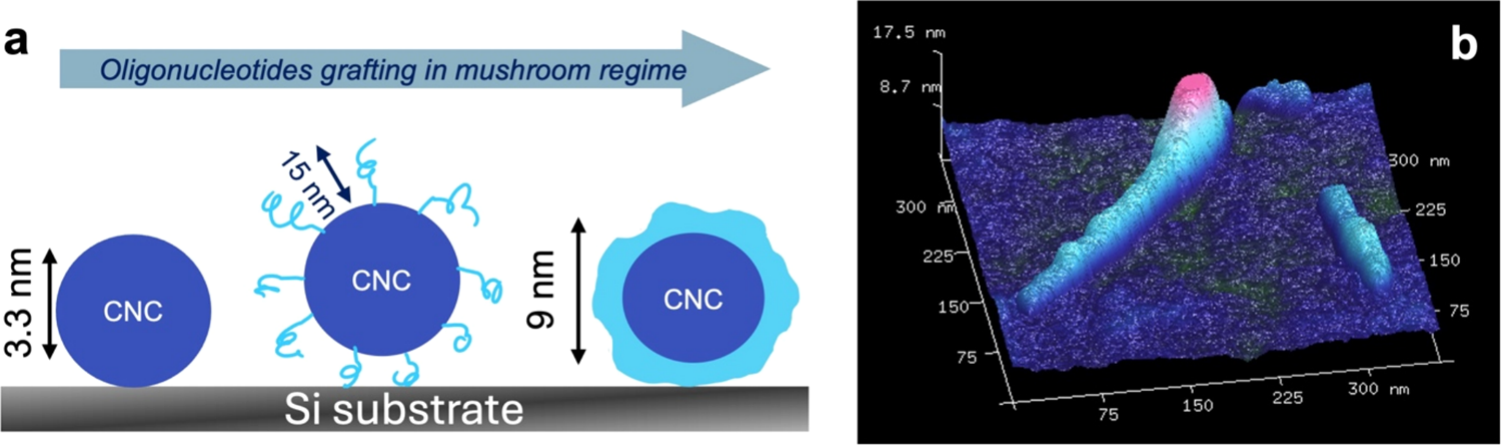
(a) Schematics of the changes in CNC dimensions according to the suggested grafting density. From left to right are the pristine CNC, initial modest grafting, and the final nonuniform “shell” of overlapped oligonucleotides in the mushroom regime. (b) 3D AFM topography image of oligonucleotide-modified nanocrystals demonstrating aggregated oligonucleotide “shell” in the dry state along the CNC visualizing nonuniform 3D surface topography.

The resulting grafted chains density was calculated to be 0.28 chains/nm^2^ or one chain per 3.5 nm^2^, and the distance between grafting sites, *D*, was calculated to be 2.2 nm from [*D* (nm) = (4/πΓ)^1/2^].^73^ This distance is approximately twice as large as the diameter of an ssDNA (1 nm). Thus, taking these numbers into consideration, and the highlighted above difference in the diameters of unmodified and grafted nanocrystals leads us to suggest that the appended DNA strands are in the mushroom regime,^74^ with a significant fraction of chains folded or bent, given the free volume available after chain end grafting (Figure 5a).

We would expect the moderate grafting density semibrush regime to be the most optimal for further CNC assembly into chiral thin films as driven by the complexation of complementary strands from neighboring nanocrystals. Indeed, in the low grafting density mushroom regime, oligonucleotides tend to aggregate on substrates. On the other hand, in a high-density brush regime, the distance and free volume between the individual strands may not be sufficient to interpenetrate and form complementary base pairs of strands between interacting nanocrystals.

Under lower grafting density, the oligonucleotides assemble more compactly on the surface of CNCs with an occasional appearance of aggregated blobs visible along the individual nanocrystals (Figure S7c). Such a nonuniform surface of the CNC-oligonucleotide can be visualized on the 3D AFM topography image and compared to the smooth surface of pristine CNCs (Figures 5b and S9). The roughness of modified CNC is also much larger than that of the substrate (0.35 ± 0.5 nm for different CNCs vs 0.2 ± 0.2 nm) for the areas between the nanocrystals (within surface areas of 100 × 100 nm^2^). Finally, the linear roughness measured along individual nanocrystals increased significantly from 0.21 ± 0.14 nm for pristine nanocrystals to 0.93 ± 0.52 nm for modified CNC-oligonucleotide.

### 3.4 Complementary DNA-Driven Assembly

**Figure 6:**
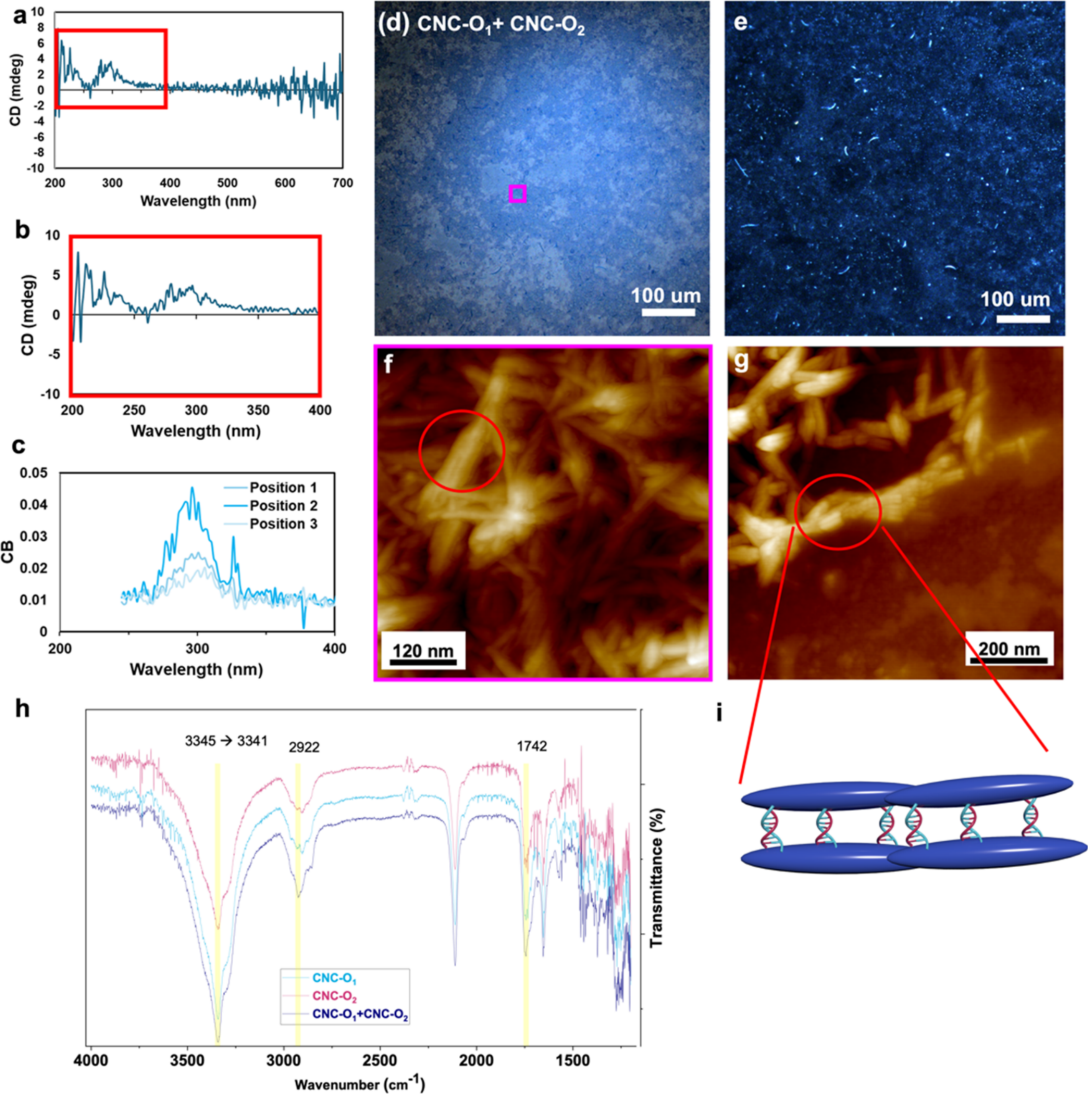
(a, b) CD spectra of the CNC-O_1_ + CNC-O_2_ assembly. (c) CB of CNC-O_1_ + CNC-O_2_ obtained from the Mueller matrix analysis of ellipsometry data, with positions 1–3 corresponding to the sample’s different rotations. Unpolarized (d) bright-field and (e) dark-field optical microscopy images of the self-assembled CNCs drop cast on the quartz slide from the mixture of CNC-O_1_ + CNC-O_2_. (f, g) AFM topography images showing CNC organization within this assembly in a solid film and of isolated nanostructures, respectively. Red circles are added to help guide the attention to the ordered nanostructures. (h) FTIR spectra of the CNCs modified with oligonucleotides before their complexation (CNC-O_1_ and CNC-O_2_) and after they form a double helix (CNC-O_1_ + CNC-O_2_). (i) Cartoon of the CNC nanostructures formed via chiral complexation of the ssDNA on their surface.

Finally, the suspensions of nanocrystals modified with complementary ssDNAs were mixed together (in the presence of 50 mM NaCl) and allowed to form a DNA duplex (dsDNA).^50,75^ The FTIR spectra of the CNCs modified with oligonucleotides (CNC-O_1_ and CNC-O_2_) were compared to the CNC mixture, referred to here as CNC-O_1_ + CNC-O_2_ to provide more details on the formation of the double helix. As DNA transitions from single strand to double helix, peak positions slightly shift, often becoming sharper and more defined in dsDNA due to the ordered structure of the double helix.^76^ The most obvious peak changes in our spectra are the N–H stretching vibrations around 3200 cm^–1^, where upon undergoing hybridization hydrogen bonding between complementary bases can cause the peaks to shift to slightly lower wavenumber and narrow, confirming the formation of the H-bonding network (Figure 6h). In this network, O–H stretching vibrations that are observed in the same wavelength range undergo the same transition, becoming narrower and shifting slightly to lower wavenumber as dsDNA is formed due to the hydration shell around dsDNA.^77^

A more rigid and ordered structure after complexation also affects the C–H stretching band around 2900 cm^–1^, causing them to shift and narrow as well.^77^ The peak around 1742 cm^–1^ corresponds to the C=O stretching that both thymine and guanine contain. As they participate in double helix formation through hydrogen bonding and base stacking, this peak is more clearly observed (Figures 6h and S10).^76^

To study how the surface modification will affect the assembly of the CNCs and their chiral interactions, the CNC-O_1_ + CNC-O_2_ mixture was further drop cast onto a quartz slide. One should expect that if the complexation is successful, the DNA duplex will show a circular dichroism of the DNA double helix.^78^ It is worth mentioning that annealing at the melting temperature of oligonucleotides (*T*_m_ = 51.9 °C) did not affect the assembly behavior and resulting properties.

Indeed, the CD spectra further confirmed complexation of the complementary strands grafted onto CNC surface into a DNA double helix. The CD spectrum of the assembled modified CNCs resembled that of a B-DNA duplex, showing a negative Cotton effect at 245 nm and a positive one at 277 nm (Figure 6a,b).^79^ The CD spectrum in solution is typically proportional to the component concentration; thus, low CD values indicate that we have a low concentration of DNA duplexes. This is not surprising, given the low concentration in suspension and the partial grafting of oligonucleotides (7 mg/mL of CNCs with ∼1 μM of oligonucleotides). As a control, the CD spectra of both CNC-O_1_ and CNC-O_2_ in DMF recorded separately and prior to DNA complexation did not display any visible peaks (Figure S11).

In polydomain thin films formed as a result of evaporation and assembly, individual tactoids can be visualized in dark-field optical microscopy (Figure 6). Optical birefringence appears as a fingerprint pattern, indicating formation of chiral nematic-type structure with helical morphology.^80^ In our case, dark-field optical microscopy of mixed CNC-DNA films showed bright microparticles reflecting a blue color (Figure 6e).

As known, circular dichroism and circular birefringence are the polarization properties characteristic of chiral CNC films.^81^ The bright blue reflection observed must be due to the birefringence of the individual anisotropic CNCs. The location and position of these optical reflection phenomena is defined by local orientation of helical domains and the local pitch length as based on the birefringence of the individual cellulose nanocrystals.^82^

Thus, taking into account that both CD and CB matrix components are manifestations of optical activity, we turned to MM ellipsometry analysis (see details in the Section 2). The analysis addresses if the birefringence of CNC-DNA materials can result in CB activity of the assembled films. Briefly, for a nondepolarizing sample, the Mueller matrix can be related (eq 1) to the circular dichroism and birefringence, CD and CB, as well as the horizontal and 45° projections of linear dichroism (LD and LD′) and birefringence (LB and LB′).^54^ Indeed, a CB peak around 300 nm was detected for the assembly, although it varied in intensity with the sample’s rotation due to linear dichroism (Figure 6c).

Thus, the detected CB activity, which is associated with helical organization, confirms that the CNCs have undergone chiral complexation of complementary oligonucleotides that were independently grafted onto the CNC surface and formed double-stranded DNA complexes between the CNCs. In addition to the observed complexation of oligonucleotides, CNCs that underwent complexation (CNC-O_1_ + CNC-O_2_) dried on the quartz slide and showed a faint blue coloration visible to the naked eye, which was also observed under optical microscopy (Figures 6d and S12). This is an additional indication for the potential of structural color preservation in films of DNA-modified CNCs.

Finally, AFM images showed randomized modified nanocrystals forming a bundle-like assembly due most likely to competing trends of local packing from cellulose nanocrystals and oligonucleotides side chains (Figures 6f,g and S13). Assembly of the CNCs driven by complementary side-chain DNA hybridization manifests in the formation of larger, bundle-like morphology of nanocrystals forming chiral nanostructures (Figure 6f,g,i).

## 4 Conclusions

In conclusion, this study demonstrates the click-chemistry approach to chemically graft complementary oligonucleotides onto CNC surfaces. Copper-free click chemistry resulted in grafting oligonucleotides onto the CNC surface, including a higher reaction yield, fast reaction rate without additional catalysts, and no by-products, thus confirming the efficacy of click chemistry in mediating covalent bonding between CNCs and functionalized ssDNAs. The observation of the initial complexation of DNA molecules in assembled CNC films illustrates the potential for chiral complexation between the nanocrystals. The observed CNC–DNA nanocomplexes demonstrated chiroptical activity and showed initial coloration as potential for structural color preservation, thus suggesting the feasibility of utilizing complementary CNC-DNA components in complex molecular assembly processes.

Our preliminary observations of the DNA complexation in solid films of DNA-modified CNCs provide valuable insights into the potential for chiral interactions between these two entities, laying the groundwork for future developments in CNC-centered complex DNA-guided assembly. We suggest that future research in this field should focus on refining surface modification strategies to achieve more uniform and complete functionalization of CNCs with DNA strands to obtain the true brush regime, as well as to increase the CNC surface charge and thus the colloidal stability needed for their long-range hierarchical organization with tailored assembly.

The results of this study suggest the feasibility of leveraging CNCs as scaffolds for the assembly of DNA-based nanostructures, opening new avenues for the development of functional, responsive, and bioderived nanostructures capable of dynamic reconfiguration. DNA complexes can exhibit different forms and structures of different handedness. Toehold-mediated strand displacement can also reverse the DNA hybridization bringing the nanocrystals together,^83^ enabling dynamic control of their assembly. We expect that DNA-grafted CNC chiroptical properties can be enriched from such unique functionalization by precisely controlling nanocrystal pairing from different oligonucleotides and in different ionic strengths or pH values.^84^ Operation under diverse environmental conditions should be explored by either working with a nonorganic solvent or a mixture of different solvents suitable for surface modifications and component stabilization (for example, DMSO–water or DMF–water mixtures). Controlling assembly with respect to guiding their complementary intermolecular interactions through selective grafting and chemical groups coupling on the surface of nanocrystals gives a specific control level of the assembly. Additionally, by changing the handle handedness and length, we expect this method to allow for a change in the CNCs overall chiral assembly with respect to the handedness and/or pitch length and, thus, a change in the position of the photonic band gap and circular polarization ability.

## Supporting Information Available

Additional experimental results including expanded characterization of modified cellulose nanocrystals via XPS, AFM, FTIR, CD, and digital photographs of the samples (PDF)
