# Supplementary material for: Click-Chemistry-Enabled Functionalization of Cellulose Nanocrystals with Single-Stranded DNA for Directed Assembly
Source: ACS Biomater Sci Eng. 2024 Sep 11;10(10):6155–66. doi: 10.1021/acsbiomaterials.4c01518 (PMC11480941; doi:10.1021/acsbiomaterials.4c01518)
Supplement: Supplementary file 1 — ab4c01518_si_001.pdf [file ab4c01518_si_001.pdf]

Supporting Information for

Click Chemistry-Enabled Functionalization of Cellulose Nanocrystals with Single-Stranded DNA for Directed Assembly

Daria Bukharina[1], Katherine Cauffiel[1], Laura Mae Killingsworth[1], Justin A. Brackenridge[1], Valeriia Poliukhova[1], Minkyu Kim[1,2], Justin Brower[3,4], Julio Bernal-Chanchavac[3,4], Nicholas Stephanopoulos[3,4], Vladimir V. Tsukruk[1*]

[1] School of Materials Science and Engineering, Georgia Institute of Technology, Atlanta, GA, 30332 USA

[2] Department of Chemical Engineering, Dankook University, Yongin, 16890 Republic of Korea

[3] School of Molecular Sciences, Arizona State University, Tempe, AZ, 85281USA

[4] Biodesign Center for Molecular Design and Biomimetics, Arizona State University, Tempe, AZ, 85251 USA

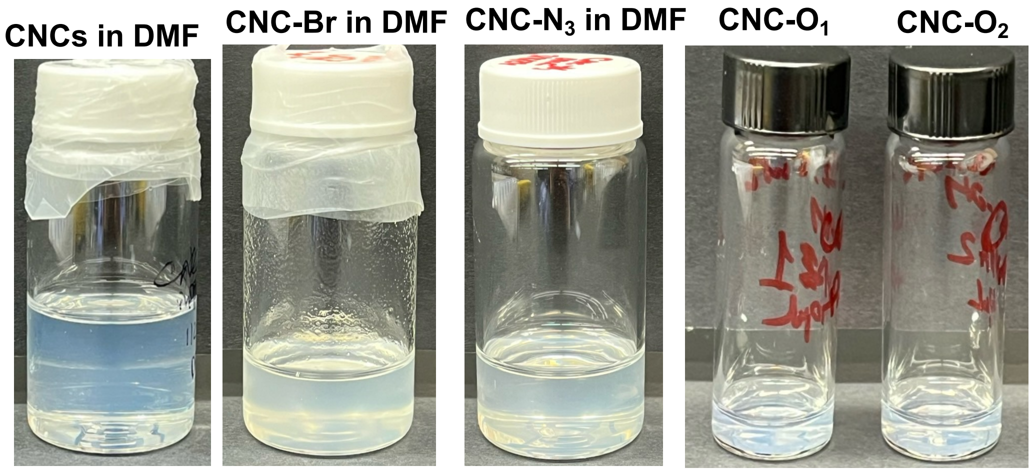

Figure S1. Digital photographs of pristine and modified CNC suspensions in DMF.

Table S1. Zeta potentials values for CNC suspensions in this study.

Sample	Zeta-potential, mV
**CNC-OH (in water)**	-52 ± 2.4
**CNC-OH (in DMF)**	-14.12 ± 12.03
**CNC-Br**	-12.8 ± 5.6
**CNC-N3**	-19.9 ± 1.6
**CNC-O1+CNC-O2**	-27.12 ± 3.1

The following strands were purchased from TriLink Biotechnologies:

Oligo$_3$ (O_3) 5’ GATTGGAAGTGGATAA{.AmC3}{DBCO} 3’

Oligo$_4$ (O_4) 5’ TTATCCACTTCCAATC{.AmC3}{DBCO} 3’

{.AmC3} indicates 3' C3 Amino Linker {DBCO} indicates DBCO-NHS.

CNCs suspensions were more stable in DMF (compared to water), presented in insets as digital photographs of CNC-O_3 and CNC-O_4. Suspensions in water did not demonstrate blue translucent coloration (seen in CNC-O_1 and CNC-O_2 in DMF) and would aggregate causing precipitation within 48 hours if left undisturbed (**Fig. S2**).

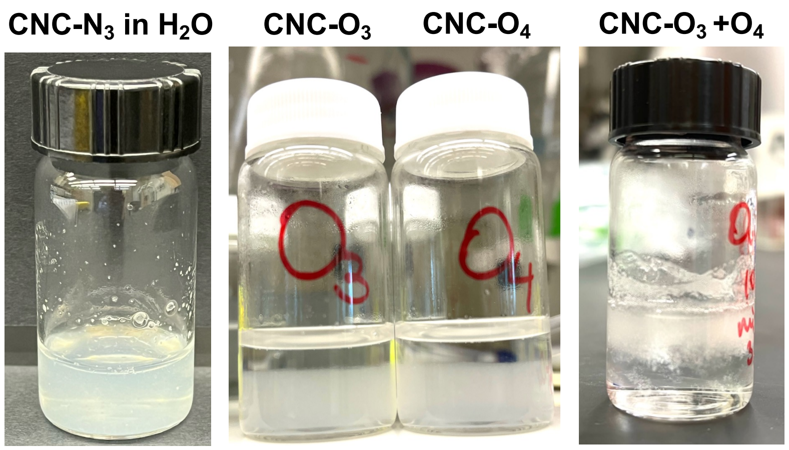

Figure S2. Digital photographs of modified CNC suspensions in water (H_2O).

Characteristically, DBCO-modified oligonucleotides showed diminished absorbance at 260 nm relative to unreacted amine-modified oligonucleotides and a corresponding increase in absorbance at 309 nm. Thus, RP-HPLC fractions were collected based on their relative increase in absorbance at 309 nm when overlaid with absorbance at 260 nm. The fractions were then pooled and washed in Nanopure water using a 3 kDa molecular weight cutoff filter (MWCO) to remove methanol and triethylammonium acetate (TEAA) prior to use.

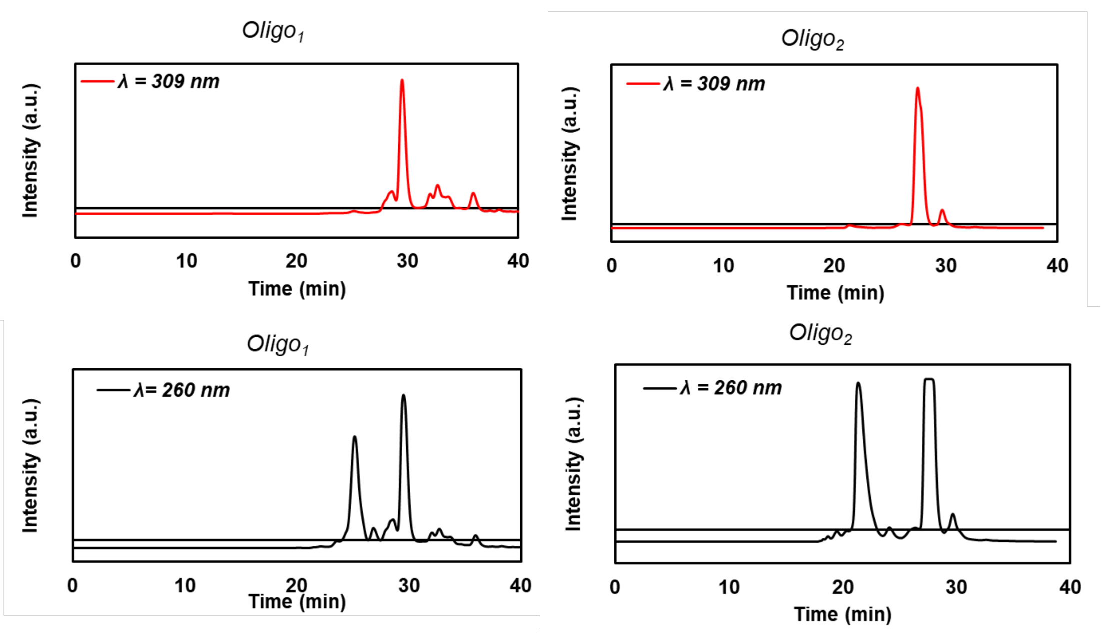

Figure S3. HPLC chromatograms of DBCO-modified DNA strands used in this study ($Oligo_1$ and $Oligo_2$).

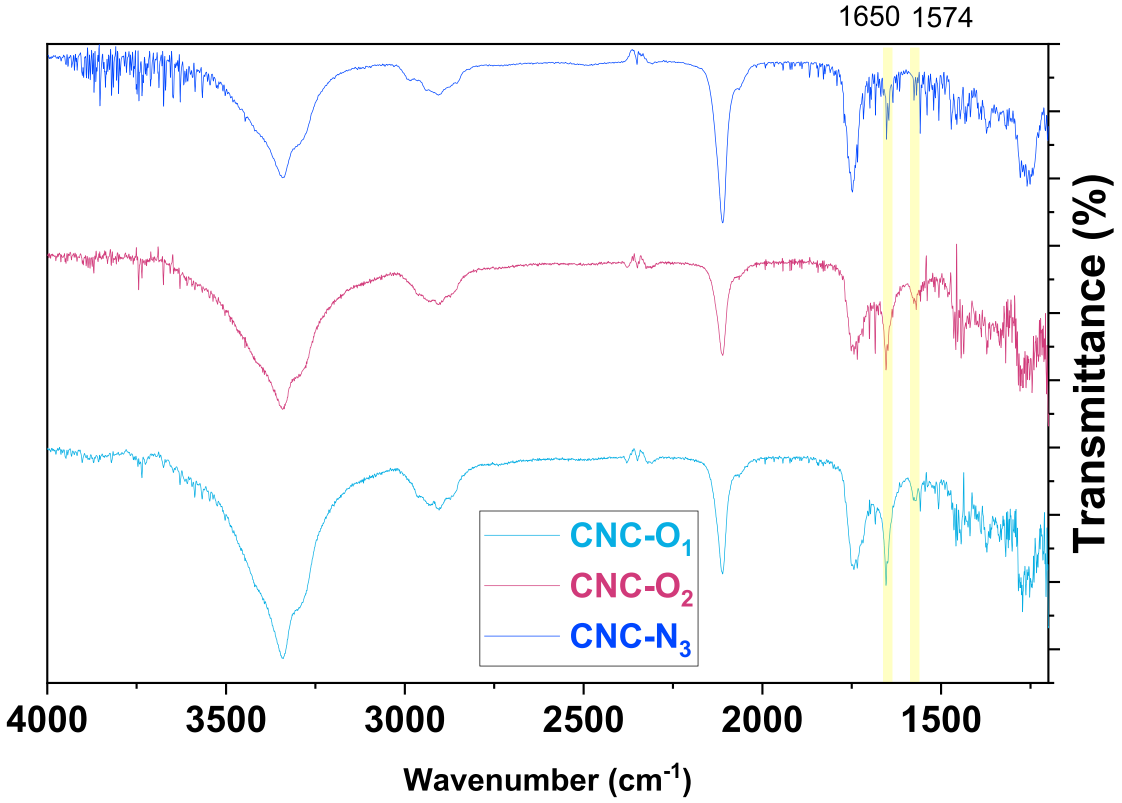

Figure S4. FTIR spectra of the CNC-N_3 and oligonucleotide modified CNCs, CNC-O_1 and CNC-O_2 as denoted.

New peaks corresponding to the formation of triazole ring appear around 1500 cm^{-1}. Aromatic rings from DBCO will show C-H stretching around 3000 cm-1 and C=C stretching around 1600 cm^{-1}. Finally, the azide peak's (2100 cm^{-1}) relative intensity decreases which serves as good indication of the triazole ring formation. Notably, samples used to record these spectra were stored in refrigerator for 6 months demonstrating stability of the surface modification overtime.

The following wide survey XPS spectra of CNC-Br showed the Br peak confirming bromination of the CNCs in addition to FTIR and UV-vis spectra (**Fig.2**). The peak at 70.8 ± 0.2 eV is characteristic of C-Br rather than contaminant species (e.g., 68.5 eV for Br^-, 67.5 eV for Br_2).[i,ii]

For CNC-N_3, it can be seen that the Br peak no longer appearing on the survey spectrum, confirming the bromine group substitution. Nitrogen peak increased in intensity and chemical bonds changes were studied in detail with XPS narrow high-resolution scans of the C 1s and N 1s described in main text.

Similarly, for CNCs modified with oligonucleotides, no bromine peaks were observed on the survey spectra and chemical bond changes resulting from click reaction were studied and detailed with XPS narrow high-resolution scans of the C 1s and N 1s described in main text.

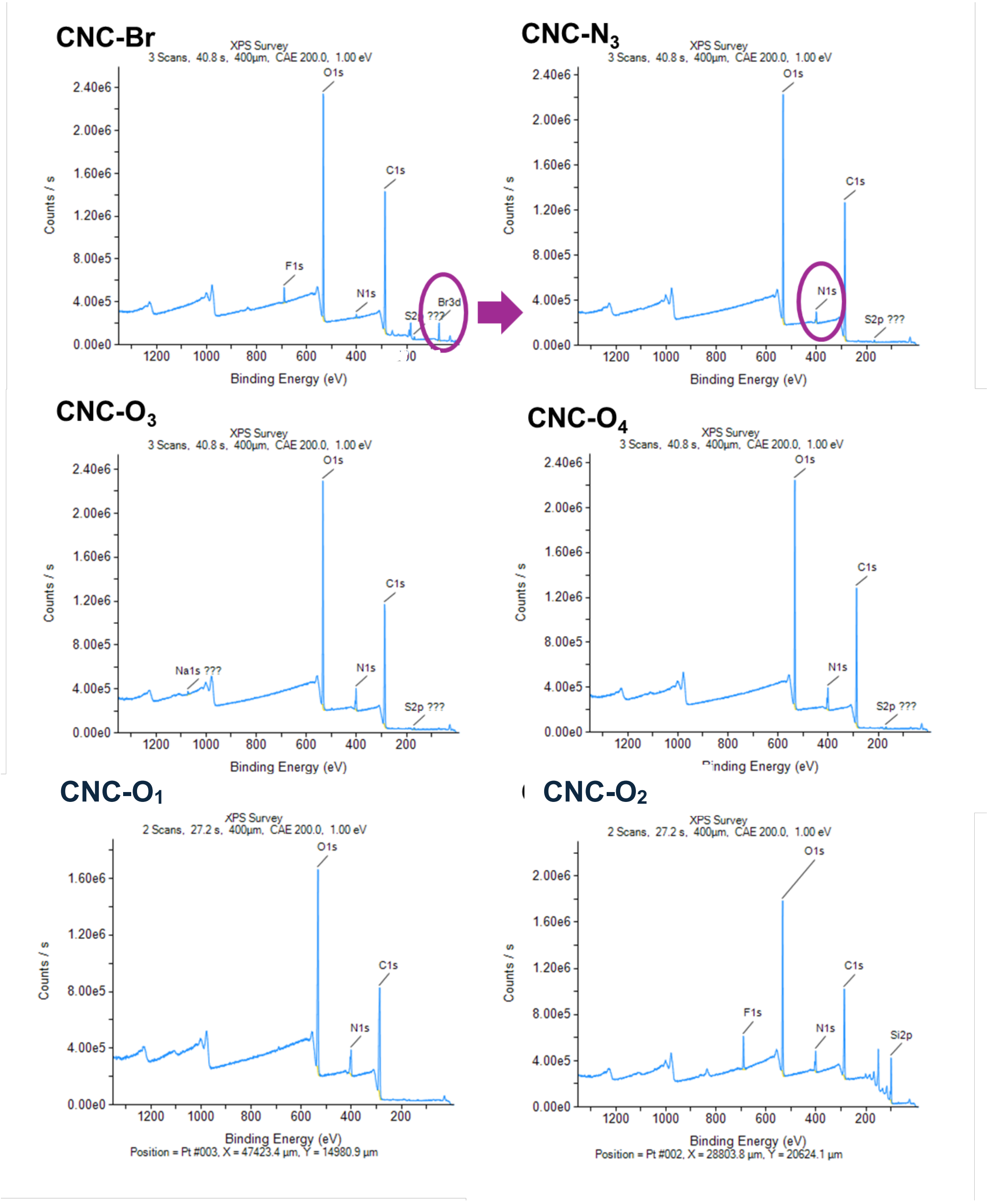

Figure S5. XPS survey scans of CNC-Br, CNC-N_3, CNC-O_3, CNC-O_4, CNC-O_1 and CNC-O_2.

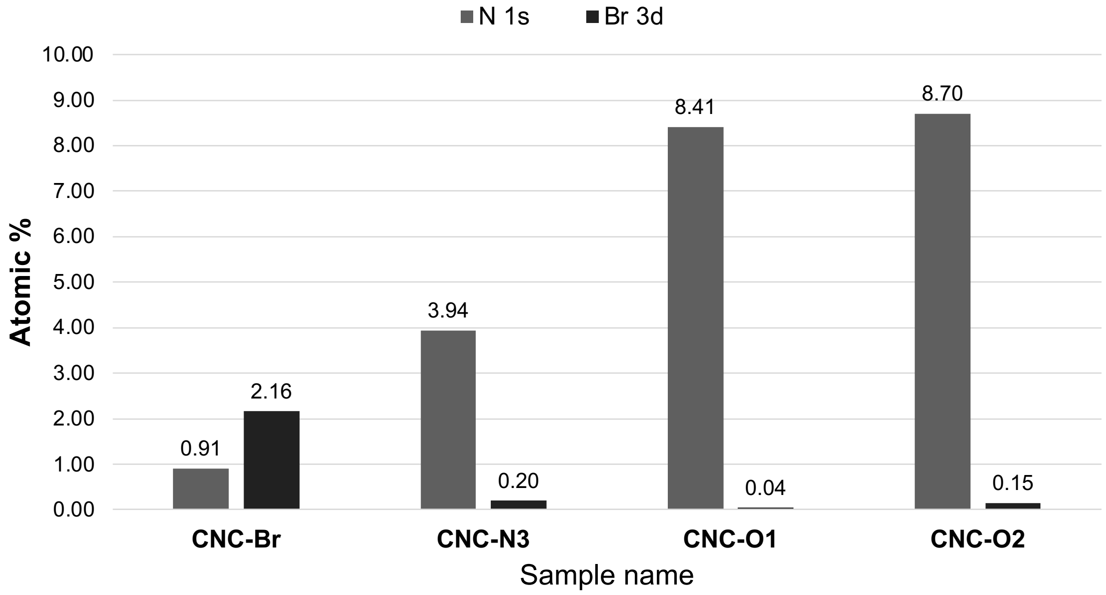

Figure S6. Atomic percent change of the N and Br elements in CNC samples during various modification steps obtained from XPS survey scans.

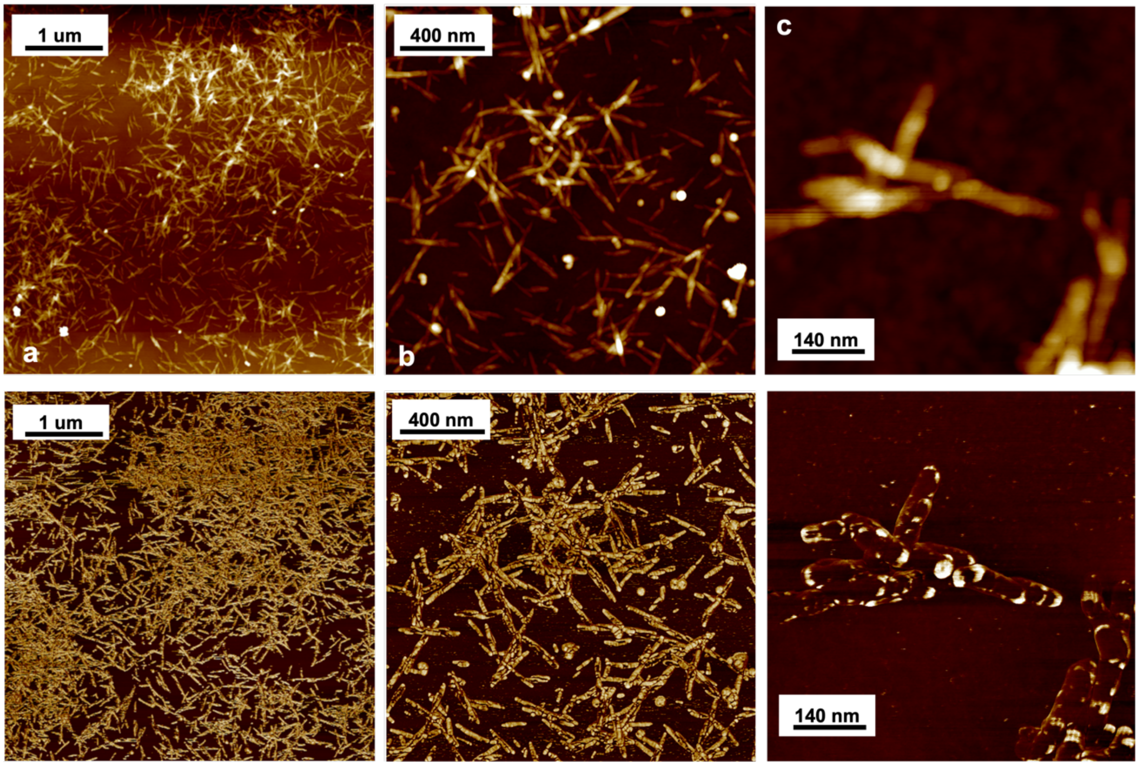

Figure S7. AFM topography (top row) and phase (bottom row) images of CNC-O_1.

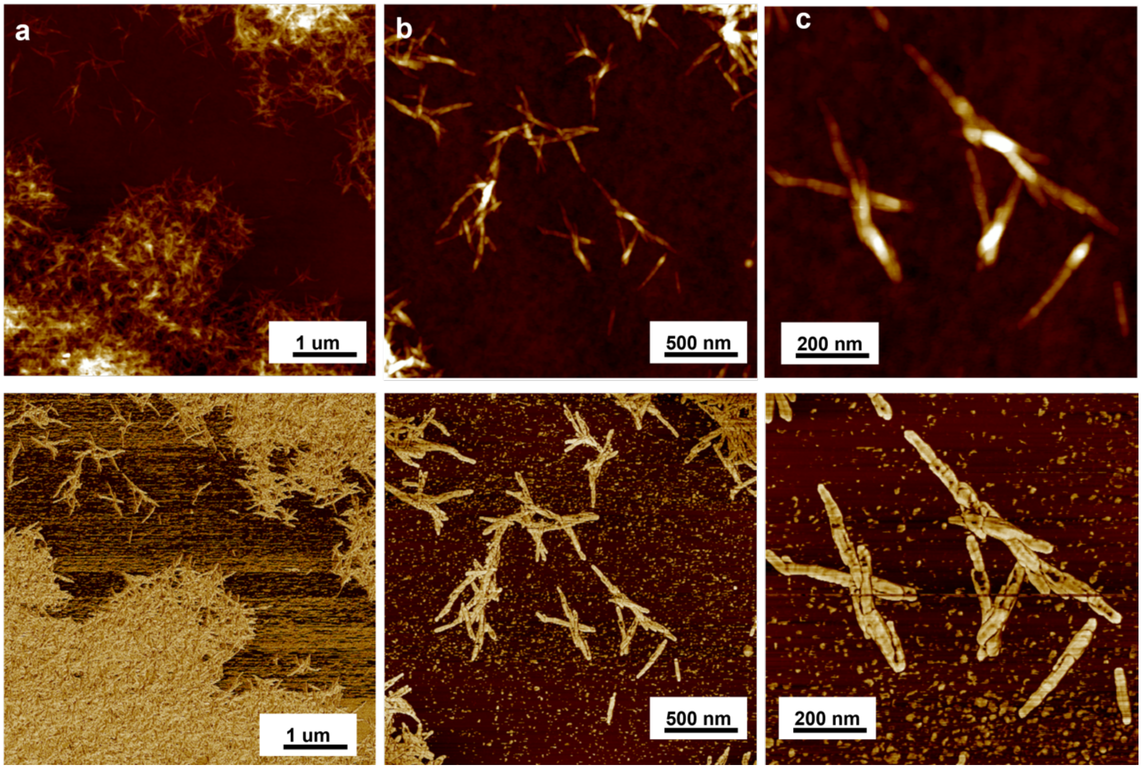

Figure S8. AFM topography (top row) and phase (bottom row) images of CNC-O_2.

The 3D AFM topography images help to visualize how ssDNAs are grafted on the CNCs surface the non-uniformly. Compared to the pristine CNC (**Fig. S9 c,f**) where their surface is smooth and uniform with the exception of height spikes dues to the nanocrystals overlap seen in topography image. For oligonucleotides-modified CNCs (CNC-O_1 and CNC-O_2), we observed that even for 'isolated' individual objects (CNCs) the surface appears patchy and non-uniform.

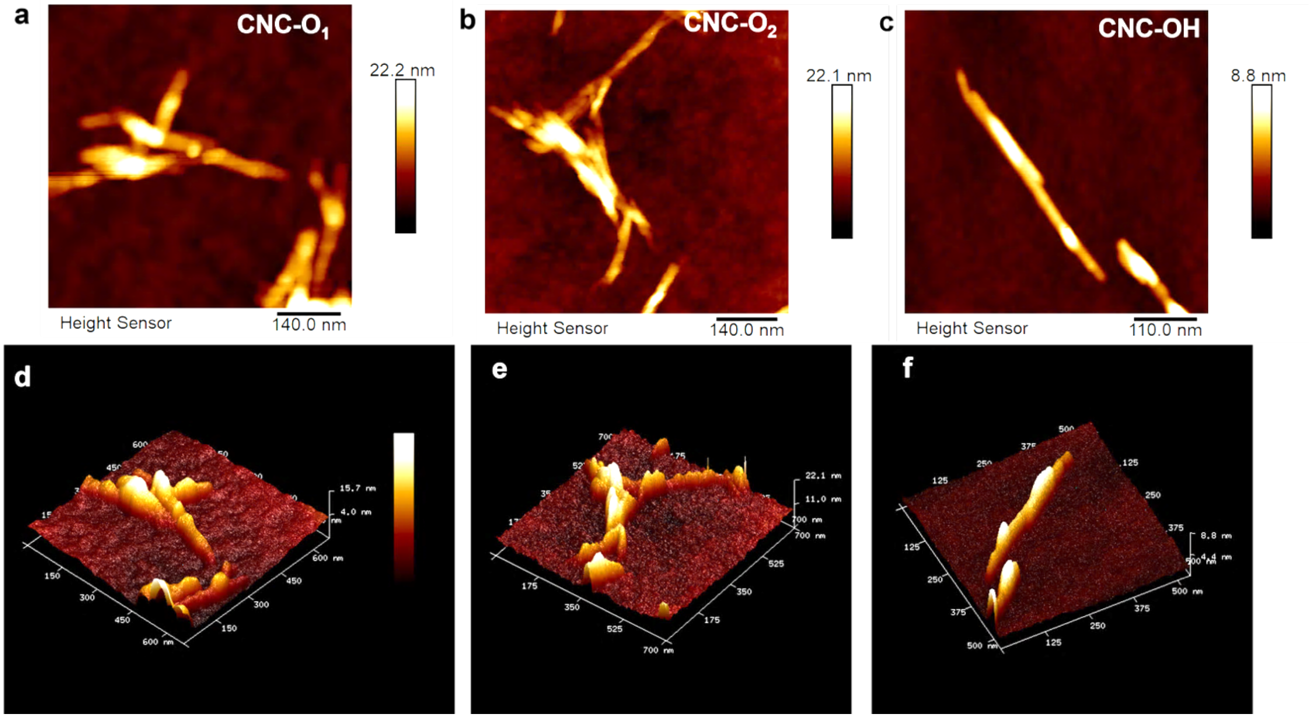

Figure S9. AFM topography images of the (a) CNC-O_1, (b) CNC-O_2 and (c) unmodified CNC-OH and corresponding to them 3D AFM topography images (d-f).

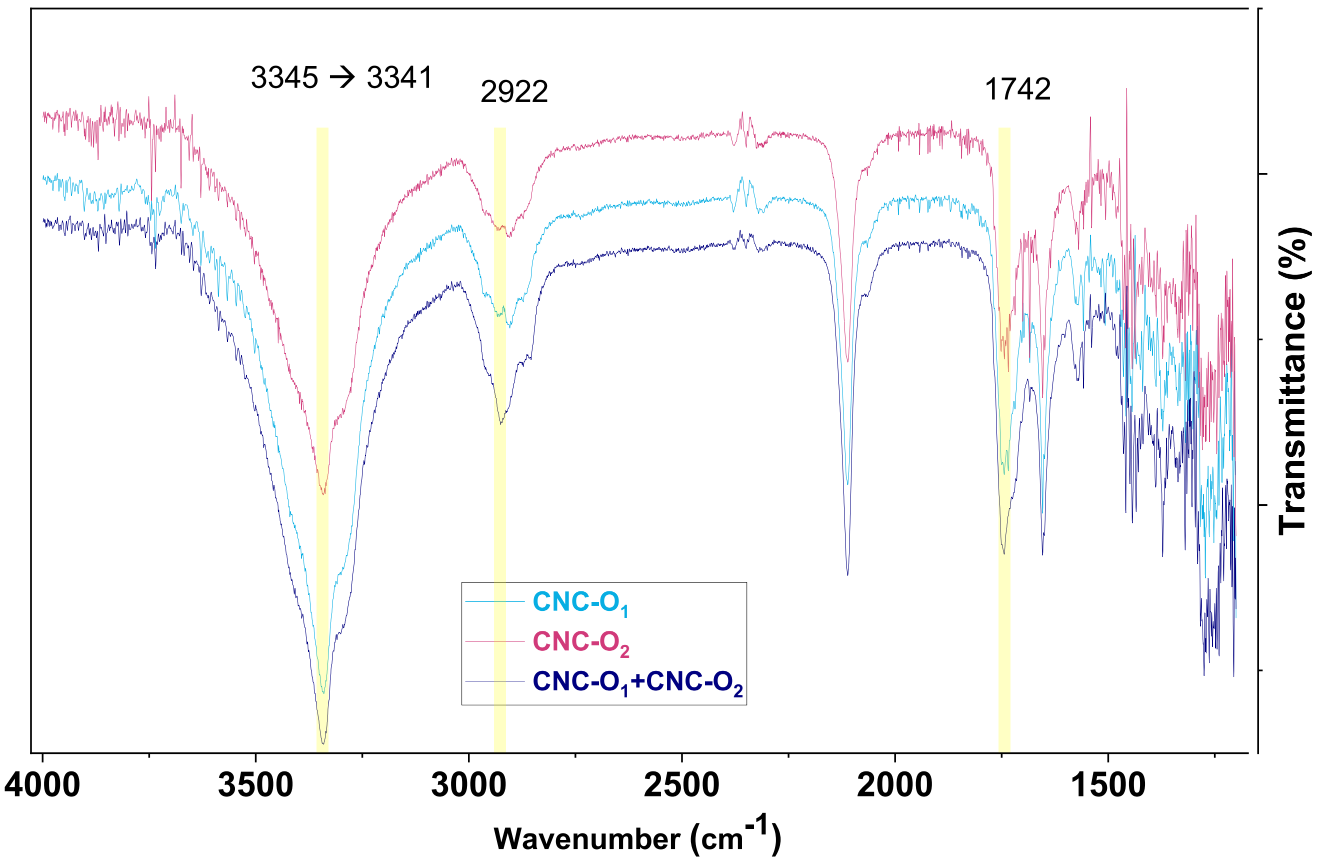

Figure S10. FTIR spectra of the CNC-O_1, CNC-O_2 and mixture of CNC-O_1+CNC-O_2.

Figure S10 is the same as Figure 6h but allows for better spectra visibility. Notably, samples used to record these spectra were stored in refrigerator for 6 months demonstrating stability of the surface modification overtime.

The CD spectra below measured for CNC-O_1 and CNC-O_2 separately prior to their complexation did not exhibit any visible peaks. The increased signal noise below 250 nm is due to strong absorbance of the DMF solvent in this wavelength region.

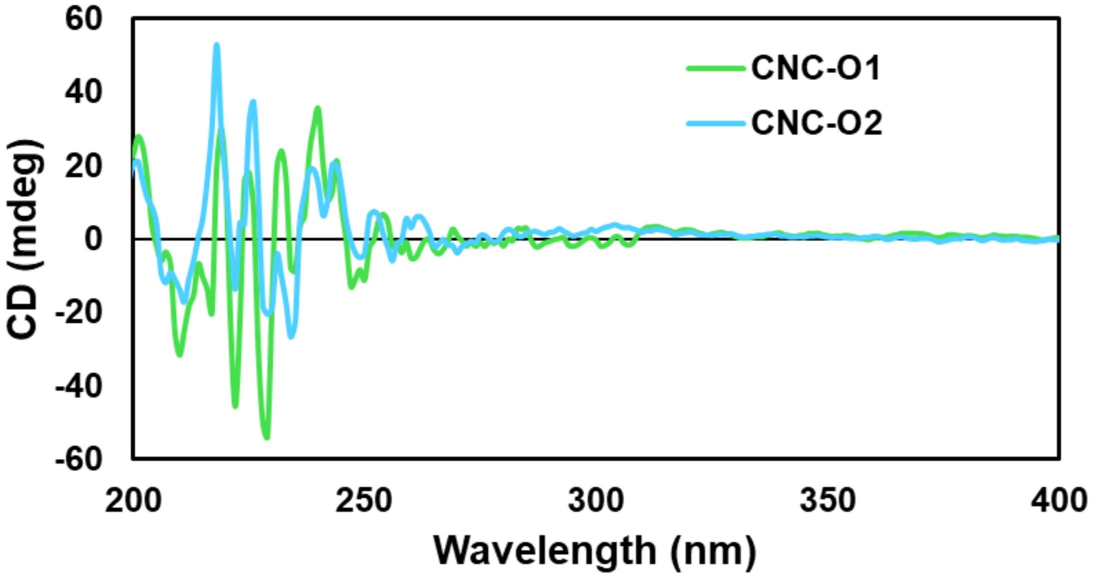

Figure S11. CD spectra of CNC-O_1 and CNC-O_2 in DMF.

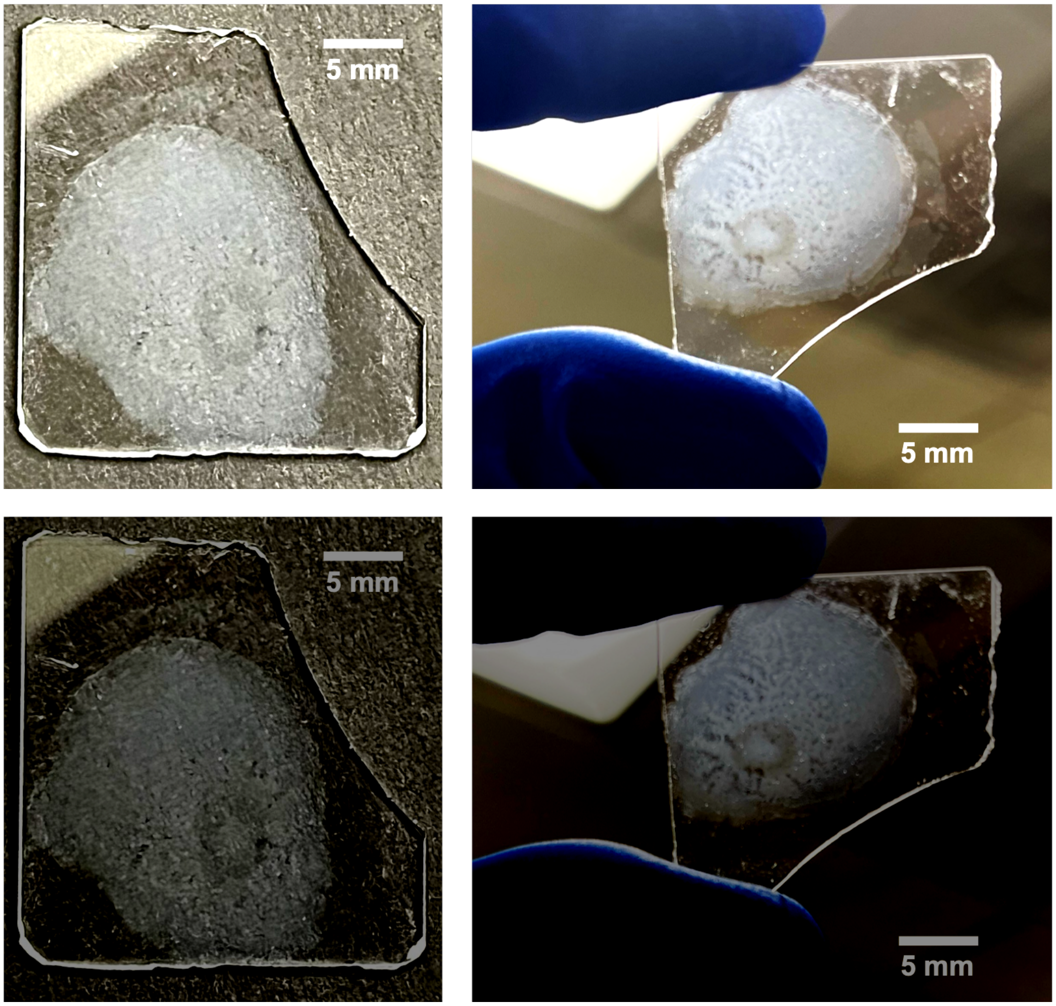

Figure S12. Digital photographs of drop casted and dried on quartz slide suspension of CNC-O_1 + CNC-O_2 undergone DNA complexation. The photographs depict faint blue coloration of the thin solid film. The photographs below are the same digital photographs of the sample but with decreased brightness to help visualize the blue coloration.

Below are the AFM topography and phase images of the-modified-with-complementary DNA-strands CNCs (CNC-O_1 + CNC-O_2) mixed in the solution state, went through chiral complexation and then drop-casted to allow them to self-organize in the solid film. The morphology of this organization was studied at different length-scales. Although, the DNA-functionalized CNCs did not exhibit long range order (**Fig.S13a**), on a smaller scale, the nanocrystals formed ordered micro-structures, bundles, seen in **Figure S13 b,c** (same as **Fig. 6f,g**).

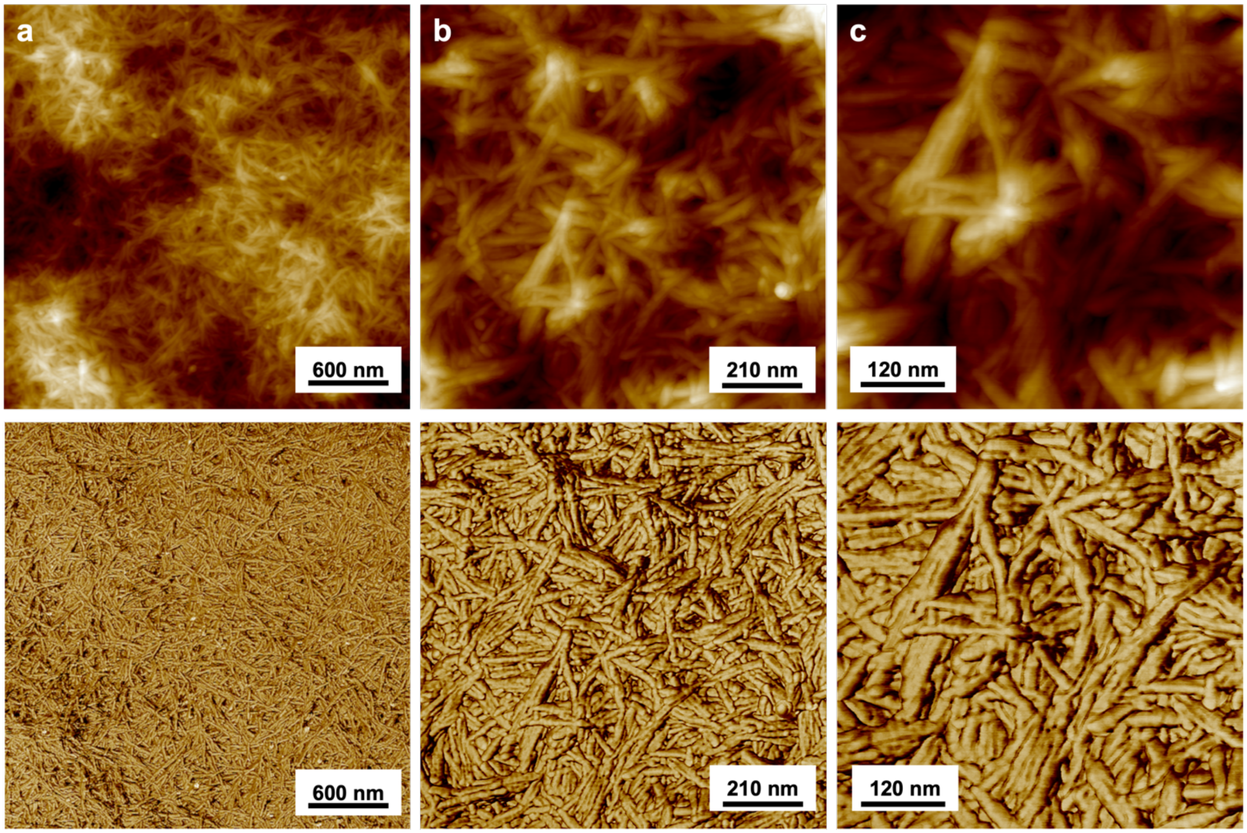

Figure S13. AFM topography (top row) and phase (bottom row) images of CNC-O_1 + CNC-O_2 drop-casted from the suspension.

We suggest these bundles to be the source of the optical activity recorded for the assembly, as they underwent the chiral complexation between DNA strands forming a double helix, in conjunction with the CNCs birefringence resulting in circular birefringence ability.

REFERENCES

[i] Zheng, J.; Liu, H.-T.; Wu, B.; Di, C.-A.; Guo, Y.-L.; Wu, T.; Yu, G.; Liu, Y.-Q.; Zhu, D.-B. Production of Graphite Chloride and Bromide Using Microwave Sparks. *Sci. Rep.* **2012**, *2* (1), 662.

[ii] Papirer, E.; Lacroix, R.; Donnet, J.-B.; Nanse, G.; Fioux, P. XPS Study of the Halogenation of Carbon Black-Part 1. Bromination. *Carbon* **1994**, *32* (7), 1341–1358.
